# Supplementary material for: Real-Time Tracking of Highly Luminescent Mesoporous Silica Particles Modified with Europium β-Diketone Chelates in Living Cells
Source: Nanomaterials (Basel). 2021 Jan 29;11(2):343. doi: 10.3390/nano11020343 (PMC7919370; doi:10.3390/nano11020343)
Supplement: Supplementary file 1 [file nanomaterials-11-00343-s001.pdf]

Supplementary Material

Real-Time Tracking of Highly Luminescent Mesoporous Silica Particles Modified with Europium β-Diketone Chelates in Living Cells

Jong-Seok Kim [1,*], Sung Ki Lee [2], Hansol Doh [3], Myeong Yun Kim [4] and Do Kyung Kim [4,*]

[1] Myunggok Medical Research Institute, College of Medicine, Konyang University, Daejeon 35365, Korea
[2] Department of Obstetrics and Gynecology, Konyang University Hospital, Daejeon 35365, Korea.; sklee0728@konyang.ac.kr
[3] Department of Food Science and Technology, University of California-Davis, Davis, CA 95616, USA; hdoh@ucdavis.edu
[4] Department of Anatomy, College of Medicine, Konyang University Hospital, Daejeon 35365, Korea; my5960@nate.com
* Correspondence: jskim7488@konyang.ac.kr (J.-S.K.); dokyung@konyang.ac.kr (D.K.K.); Tel.: +82-42-600-8648 (J.S.K.); Tel.: +82-42-600-6445 (D.K.K.)

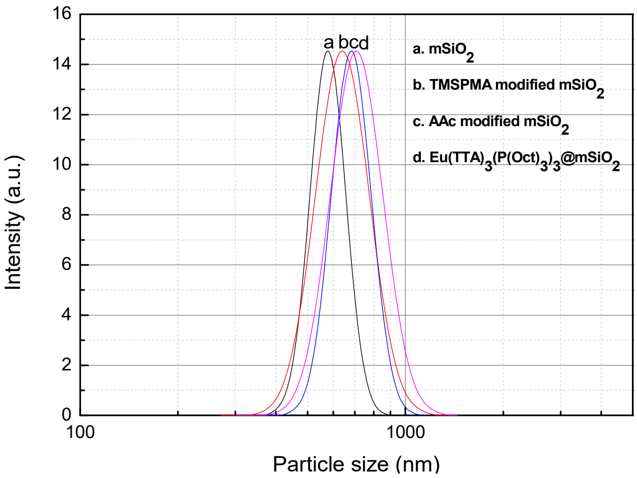

Figure S1. Normalized particle size distribution curves of step-by-step modified samples for (a) $mSiO_2$; (b) TMSPMA modified $mSiO_2$ (TMSPMA@$mSiO_2$); (c) MMA modified $mSiO_2$ (MMA@$mSiO_2$) and (d) $Eu(TTA)_3(P(Oct)_3)_3$@$mSiO_2$.

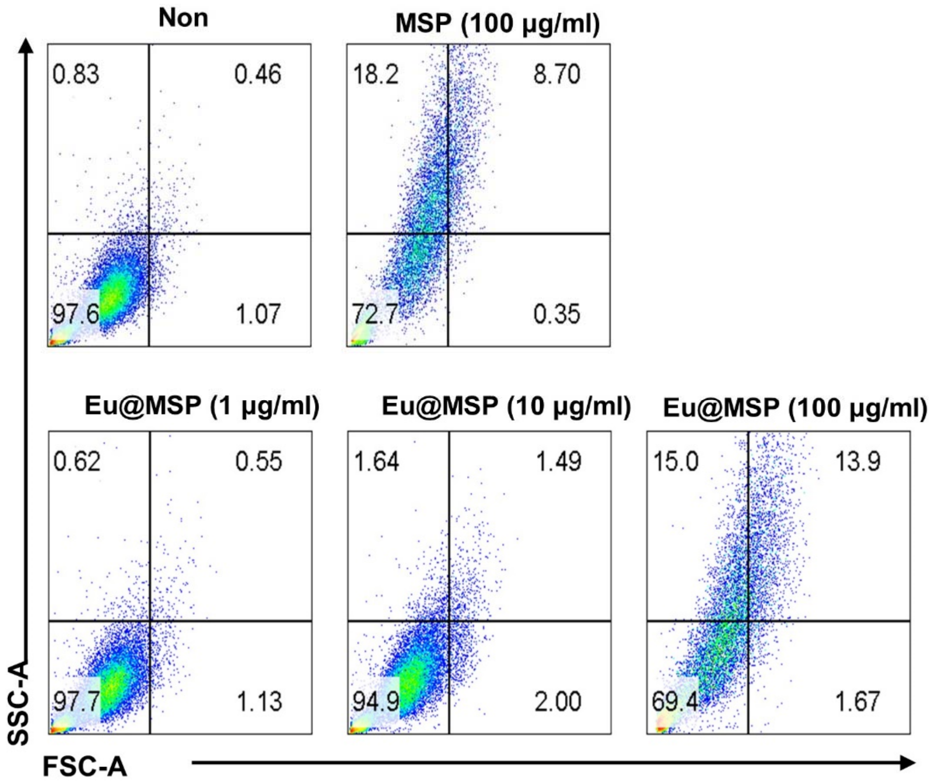

Figure S2. Measuring the size and internal complexity/granularity change of cells by MSP and Eu@MSP uptake using their scatter properties by flow cytometry.
